# Oligomeric state, hydrodynamic properties and target recognition of human Calcium and Integrin Binding protein 2 (CIB2)

**DOI:** 10.1038/s41598-019-51573-3

**Published:** 2019-10-21

**Authors:** Giuditta Dal Cortivo, Valerio Marino, Claudio Iacobucci, Rosario Vallone, Christian Arlt, Anne Rehkamp, Andrea Sinz, Daniele Dell’Orco

**Affiliations:** 1 Department of Neurosciences, Biomedicine and Movement Sciences, Section of Biological Chemistry, University of Verona, Verona, Italy; 2 Department of Translational Research and New Technologies in Medicine and Surgery, University of Pisa, Pisa, Italy; 3 Department of Pharmaceutical Chemistry and Bioanalytics, Institute of Pharmacy, Charles Tanford Protein Center, Martin Luther University Halle-Wittenberg, Halle, Germany; 4 Structural Biology and Biophysics Unit, Fondazione Ri.MED, Palermo, Italy

**Keywords:** Biochemistry, Biophysics, Chemical biology

## Abstract

Calcium- and Integrin-Binding protein 2 (CIB2) is a small and ubiquitously expressed protein with largely unknown biological function but ascertained role in hearing physiology and disease. Recent studies found that CIB2 binds Ca^2+^ with moderate affinity and dimerizes under conditions mimicking the physiological ones. Here we provided new lines of evidence on CIB2 oligomeric state and the mechanism of interaction with the α7B integrin target. Based on a combination of native mass spectrometry, chemical cross-linking/mass spectrometry, analytical gel filtration, dynamic light scattering and molecular dynamics simulations we conclude that CIB2 is monomeric under all tested conditions and presents uncommon hydrodynamic properties, most likely due to the high content of hydrophobic solvent accessible surface. Surface plasmon resonance shows that the interaction with α7B occurs with relatively low affinity and is limited to the cytosolic region proximal to the membrane, being kinetically favored in the presence of physiological Mg^2+^ and in the absence of Ca^2+^. Although CIB2 binds to an α7B peptide in a 1:1 stoichiometry, the formation of the complex might induce binding of another CIB2 molecule.

## Introduction

Calcium- and Integrin-Binding protein 2 (CIB2) is a small (21.7 kDa) Ca^2+^ and Mg^2+^-binding protein initially discovered as a DNA-dependent protein kinase interacting protein^1^. It is a member of the CIB family, which contains homolog EF-hand proteins showing evolutionary relationship with the class of neuronal calcium sensor proteins^2^. Its broad expression levels in a variety of tissues suggests the direct involvement of CIB2 in a heterogeneous group of physiological and disease-associated processes, which include congenital muscular dystrophy type 1A^3^, promotion of HIV-viral infection^4^, N-methyl-D-aspartate receptor-mediated Ca^2+^ signaling in cultured hippocampal neurons^5^, and very recently sphingosine kinase 1-mediated oncogenic signaling in ovarian cancer^6^. While its physiological role remains largely unknown, some general functional features of CIB2 have been established: (i) CIB2 can bind Ca^2+^ and Mg^2+^ ions via two functional EF-hand motifs, namely EF3 and EF4, therefore switching to a specific conformation^7,8^; (ii) CIB2 specifically binds to the cytoplasmic domain of integrin α7B^3,7^, as well as to αIIb integrin^7^, thus being presumably involved in a variety of signal transduction processes.

In spite of its evidently broad biological role, recent findings suggest that CIB2 is deeply involved in hearing physiology. CIB2 knockout mice showed abolished mechanoelectrical transduction in auditory cells leading to profound hearing loss^9^, moreover missense mutations in the gene encoding for CIB2 have been found to be associated with hereditary non-syndromic deafness (DFNB48) and possibly Usher Syndrome type 1J, a genetic disorder characterized by hearing loss and progressive blindness^10,11^. Altogether, these recent findings suggest that CIB2 is an essential component for the normal development of hair cells.

**Figure 1:**
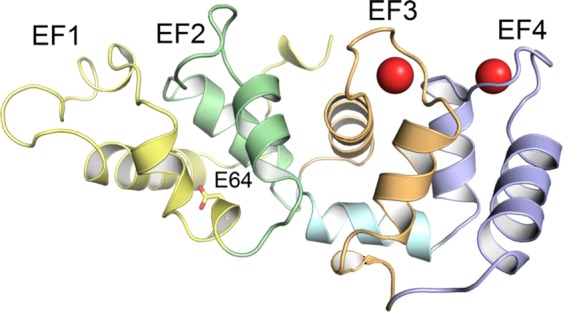
Three-dimensional structure of the homology model of Ca^2+^-bound human CIB2. Protein secondary structure is shown as cartoons, EF1 is colored in yellow, EF2 is colored in green, EF3 is colored in orange, EF4 is colored in blue, the C-terminal helix is colored in cyan and Ca^2+^ ions are shown as red spheres. Residue E64 is shown as sticks. The structural model is from ref.^8^ (see Methods).

In a recent study^8^ we investigated the capability of CIB2 to bind Ca^2+^ and Mg^2+^ thereby switching to a functionally distinct structural state. By integrating several biochemical and biophysical techniques we could establish that in the absence of any cation CIB2 forms a molten globule state that essentially lacks tertiary structure. Binding of either Ca^2+^ or Mg^2+^ leads to a highly similar tertiary structure although the apparent affinity for Ca^2+^ (K_d_^app^ = 0.5 mM) was found to be significantly lower compared to that for Mg^2+^ (K_d_^app^ = 290 μM). This is quite unusual for Ca^2+^-sensor proteins, but it could be explained by the relatively low sequence similarity in key functional regions when comparing CIB2 with the homologue CIB1 and other members of the CIB family^8^. We also found that the binding of cations to the functional motif EF3 at the C-terminal domain (Fig. 1) triggers a conformational transition that puts the EF3 loop in allosteric communication with the N-terminal domain, specifically with residue E64, that has been found mutated into an aspartic acid in Usher Syndrome type 1J^11^. Surprisingly, the apparently conservative E64D mutation abolishes the conformational switch and significantly lowers the affinity for both cations^8^, indicating that even a small difference such as the presence or absence of a methylene bridge between acidic amino acids can be critical to whether an essential interaction can take place or not. Both wild type (WT) and E64D-CIB2 were found to recognize and bind a peptide from the known target integrin α7B^3,7^ with similar affinity in the micromolar range, and based on analytical size exclusion chromatography (SEC), dynamic light scattering (DLS) and non-denaturing electrophoresis assays we concluded that CIB2 is dimeric, in line with independent previous observations^11,12^.

In an attempt to define the structural features of the CIB2 dimeric interface and its interaction with putative target peptides by employing cross-linking/mass spectrometry^13–15^, we found evidence of monomeric CIB2, but surprisingly no evidence of dimers. In this work we therefore sought to thoroughly characterize the oligomeric state of CIB2 and the interaction with its established target. A variety of mass spectrometry (MS)-based techniques, including native ESI-MS, MALDI-TOF-MS, and cross-linking/MS (XL-MS) experiments of the intact protein, were integrated with novel SEC and DLS experiments based on a more accurate selection of the heterogeneous components of the elution bands. The analysis of the newly assessed hydrodynamic properties of CIB2, in comparison with those of two other Ca^2+^-sensors, namely calmodulin (CaM) and recoverin (Rec), helped explaining the misinterpretation in our previous results as to the dimeric nature of CIB2, thus calling for particular caution when using analytical SEC for assessing the apparent molecular weight of Ca^2+^-sensor proteins. The MS-based techniques as well as surface plasmon resonance (SPR) analysis exclude dimerization of CIB2 over the broad range of conditions tested in this study, and highlight a specific interaction only with the membrane proximal segment of the cytoplasmic domain of α7B integrin, in line with previous results by us^8^ and others^7^, revealing no significant interaction with the C-terminus proximal region.

## Results and Discussion

### Native Mass Spectrometry and XL-MS analyses do not show evidence of CIB2 dimers

**Figure 2:**
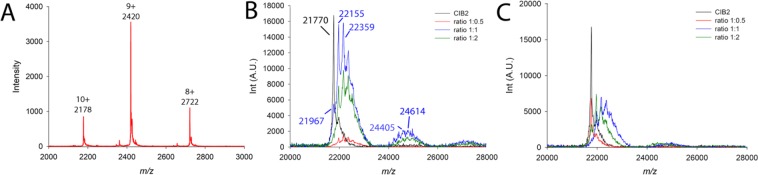
Results from MS experiments. (**A**) Native ESI mass spectra collected in the presence of 14 µM CIB2 in 200 mM ammonium acetate, pH 6.8. Collision energy was set to 60 V. (**B**,**C**) MALDI-TOF-MS measurements of cross-linked CIB2 samples with peptides α7B_M (**B**) and α7B_Scrb (**C**). 10 µM CIB2 was cross-linked with 50-fold molar excess of DSBU with increasing concentrations (5 μM, red line; 10 μM, blue line; 20 μM, green line) of α7B_M (**B**) and α7B_Scrb (**C**) peptides in the presence of 1 mM Mg^2+^ and 2 mM Ca^2+^. Between 1000 to 3000 laser shots were accumulated to one MALDI-TOF mass spectrum. CIB2 without DSBU cross-linker was included as internal control (black line).

Native electrospray ionization (ESI)-MS served to investigate the oligomeric state of CIB2. Figure 2A shows that under native pH conditions CIB2 appears in the three main charge states 8+ to 10+ with signals at *m/z* 2178, 2420, and 2722. Deconvolution of the mass spectrum yielded a molecular mass of 21,767 Da, which is in perfect agreement with the mass of 21.7 kDa. Strikingly, no signals of CIB2 dimers, nor higher order oligomers were detected in native MS experiments.

In addition, CIB2 was subjected to chemical cross-linking with DSBU (disuccinimidyl dibutyric urea) in the presence of the two peptides α7B_M and α7B_Scrb (Fig. 2B,C). While peptide α7B_M is supposed to bind to CIB2, the scrambled peptide α7B_Scrb should not show complex formation with CIB2. CIB2 appears in the MALDI-TOF mass spectrum at *m/z* 21,770 **(**Fig. 2B, black line). With increasing amount of peptide α7B_M, a 1:1 CIB2/peptide complex appears in the mass spectra, as is visible from the signals between *m/z* ~ 24,000 and 26,000 (Fig. 2B). The calculated mass of a 1:1 CIB2/peptide complex with one cross-linker molecule is 23,964 Da. The mass spectrum indicates the additional attachment of two to five cross-linker molecules to the 1:1 complex where the cross-linker DSBU has either reacted at both sides or is partially hydrolyzed. A weak signal for a 1:2 CIB2/α7B_M peptide complex is also visible in the mass spectrum (signals between *m/z* 26,500 and 28,000). Moreover, CIB2 was detected without peptide, but decorated with one to five DSBU cross-linker molecules. As such, the signal at *m/z* 21,967 corresponds to CIB2 that is intramolecularly cross-linked with one DSBU molecule (Δm = 196 u).

In contrast, cross-linking between CIB2 and peptide α7B_Scrb showed only minor signals for a 1:1 complex (Fig. 2C), but CIB2 was mainly found to be modified with one to five DSBU molecules (signals between *m/z* ~ 22,000 and 23,000).

Given that native ESI-MS only showed a CIB2 monomer and furthermore, in cross-linking experiments, a 2:1 CIB2 and peptide α7B_M complex was never observed, this clearly points to monomeric rather than dimeric CIB2.

### Analytical size exclusion chromatography and dynamic light scattering of CIB2, CaM and Rec highlight different hydrodynamic properties

**Figure 3:**
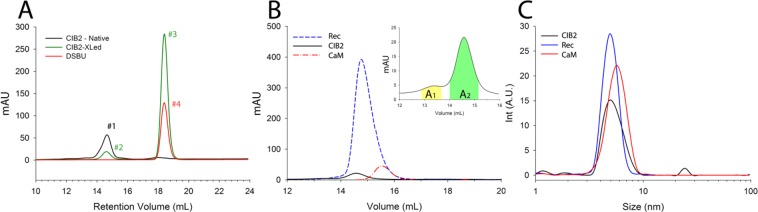
Investigation of apparent MW of CIB2 including other two Ca^2+^-sensor proteins by analytical SEC (**A**,**B**) and DLS (**C**). (**A**) MW investigation of CIB2 before (black line) and after (green line) modification with DSBU. One reaction was performed in the presence of sole DSBU (red line). (**B**) CIB2 (∼70 µM), Rec (∼80 µM) and CaM (∼110 µM) were loaded into an analytical SEC column previously equilibrated with 20 mM HEPES pH 7.4, 150 mM KCl, 1 mM DTT, 1 mM Mg^2+ ^, 2 mM Ca^2+^. The inset shows the zoom of CIB2 elution profile that was collected in two different fractions, represented by the colored areas. (**C**) The hydrodynamic diameters of the eluted peaks were assessed by DLS: 15 to 30 measurements were collected for each sample at 37 °C, consisting in at least 13–15 repetitions. The presented curves represent the average distribution. The protein concentration in the eluted fractions was found to be: 11 µM (Rec), 7.4 µM (CaM) and 1.8 µM (CIB2).

Previous analytical SEC experiments of CIB2 under reducing conditions displayed elution profiles corresponding to an apparent MW in the 37–39 kDa range in different cation-bound states, thus compatible with a dimer and incompatible with a monomer^8^. Moreover, DLS experiments performed with the samples collected immediately after elution from the SEC column measured a relatively high hydrodynamic diameter (d^Mg^ = 8.43 nm and d^Ca^ = 8.18 nm), which was also interpreted as an evidence of a dimeric protein^8^. Since the present MS-based data appear to be in clear contradiction with previous findings, we repeated both analytical SEC and DLS using identical conditions as for the XL-MS experiments. Results are reported in Fig. 3.

In the presence of 1 mM Mg^2+^, 2 mM Ca^2+^ and DSBU cross-linker (same conditions as those used in XL-MS experiments) CIB2 showed a SEC elution profile (Fig. 3A, green curve) that substantially overlapped with that of the protein without cross-linker **(**Fig. 3A, black curve) when the first elution peak is concerned (see peaks #1 and #2). According to a calibration curve obtained with globular proteins of known MW, these bands would correspond to an apparent MW (MW^SEC^) of 35.9 kDa in the absence and of 36.7 kDa in the presence of the cross-linker, thus very similar to those obtained in our previous work under slightly different conditions. The second prominent peak at higher elution times observed for cross-linked CIB2 (Fig. 3A, green curve, peak #3) corresponds to the elution of DSBU compounds, as proved by the complete overlapping with the band obtained when eluting DSBU alone (red curve, peak #4). The chemical nature of the eluate was further confirmed by NMR spectroscopy (data not shown). Therefore, although an MW^SEC^ of 37–39 kDa previously drove us to the conclusion that a protein with actual MW of 21.6 kDa eluted as a dimer, our MS and XL-MS results clearly show that the interpretation was not correct in the case of CIB2, which remained monomeric in all tested conditions.

To further investigate the nature of the misleading result we performed analytical SEC of two other Ca^2+^-sensors of similar size in the same conditions in which CIB2 was shown to elute with a significantly higher MW than that expected for a monomer. For this purpose, we used CaM (16.8 kDa) and Rec (23.3 kDa), which are both well characterized, Ca^2+^-sensor proteins. The three-dimensional structure of the three proteins was also used to estimate the theoretical value of the hydrodynamic diameter by the HydroPro software^16^ and compare it with that measured by DLS right after the elution from the SEC column. Quantitative results are reported in Table 1.

**Table 1:** Comparison of the hydrodynamic properties of CIB2, CaM and Rec.

	HydroPro Ø (nm)	Gyration radius (nm)	DLS Ø [n] (nm)^a^	Hydrophobic SAS area (nm^2^)	Hydrophilic SAS area (nm^2^)	Total SAS area (nm^2^)	MW (kDa)	MW^SEC^ (kDa)
CIB2 (13–184)^b^	4.736	1.88	5.4 ± 0.2 [15]	59.94	55.34	115.38	21.3	37.8 (A_2_ band)
Rec (1-201)^b^	5.112	1.94	5.0^a^ ± 0.1 [18]	62.54	64.68	127.23	23.3	33.5
CaM (1–149)^b^	5.076	2.22	5.8 ± 0.1 [30]	50.28	54.97	105.26	16.8	20.5

^a^Data are reported as mean ± SEM; n represents the number of measurements used for the calculation.

^b^Sequence coverage of the structural models used for hydrodynamic diameter calculation.

SEC elution profiles (Fig. 3B) showed a main elution peak for each Ca^2+^-sensor, which corresponds to an MW^SEC^ of 37.8 kDa for CIB2, 33.5 kDa for Rec and 20 kDa for CaM (Table 1). A closer look at the elution profile of CIB2, however, showed the presence of a lower intensity shoulder at lower elution time (Fig. 3B; inset). This shoulder (here denominated A_1_) was collected together with the main peak (A_2_) in our previous work^8^. In contrast, the two fractions were separately collected and analyzed in the present study. Interestingly, the effect of A_1_, with a predicted MW^SEC^ of 81.3 kDa was that of rendering the colloidal suspension analyzed by DLS more disperse and to confer CIB2 an apparently higher hydrodynamic radius. Indeed, when A_2_ was analyzed by DLS as separated from A_1_ (Figs 3C and S1), a lower hydrodynamic diameter was determined (5.4 ± 0.2 nm; Table 1 and Fig. S1), which was similar to that of Rec (5.0 ± 0.1 nm; Table 1 and Fig. S1) and CaM (5.8 ± 0.1 nm; Table 1 and Fig. S1), and significantly lower compared to that previously measured for CIB2 when both A_1_ and A_2_ were collected in one sample (d^Mg^ = 8.4)^8^. We thus conclude that our inferences as to the dimeric nature of CIB2 based on previous DLS experiments were affected by the presence of the A_1_ band, as current DLS data based on a more accurate selection of the elution peak confirm the monomeric form of CIB2.

**Figure 4:**
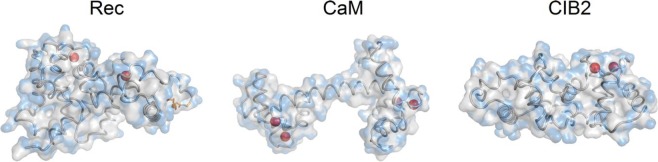
Three-dimensional structures of bovine Rec (left), human CaM (center) and human CIB2 (right) used for hydrodynamic diameter estimation. Proteins secondary structure is shown as grey tubes, Ca^2+^ ions are represented as red spheres, myristoyl group is shown as orange sticks, hydrophilic and hydrophobic solvent accessible surfaces are represented in transparency as blue and grey surfaces, respectively.

An apparent joint feature of Rec and CIB2 is that their estimated MW^SEC^ is significantly higher than that of a monomer (Table 1). Although Rec has been shown to dimerize in the presence of Ca^2+ ^^17^, dimers were clearly isolated at protein concentrations above 100 μM, so we assume that in our experiments Rec was mostly monomeric. We speculate that this fact could be related to the significantly higher hydrophobic solvent-accessible surface of Rec and CIB2 compared to that of CaM (60–62 nm^2^ vs 50 nm^2^, Table 1; Fig. 4). Solvation of their large hydrophobic surfaces might influence the interaction of CIB2 and Rec with the column matrix and result in lower retention time, that is higher MW^SEC^ compared to CaM. On the other hand, DLS data show a significantly better correlation between the measured and the theoretical hydrodynamic diameter estimated by HydroPro, especially considering that the structural model used for CIB2 was missing the first 12 residues (Table 1). The size measured by DLS seems to correlate to some extent with the radius of gyration of the protein, which accounts for molecular geometry and shape and the space distribution of amino acids. In spite of its lower MW, due to its elongated form (Fig. 4) CaM has a significantly higher radius of gyration (2.22 nm) compared to Rec (1.94 nm) and CIB2 (1.88 nm), which is reflected by a significantly higher size detected by DLS (Table 1).

Although analytical SEC has been widely used to determine the molecular weight of proteins by comparing elution volume parameters with those of different known calibration standards, the elution profile of proteins is closer to their Stokes radius rather than to their effective molecular weight, and particular care should be taken when the shape of the protein is different from the generally globular shape of the standards^18^. For Ca^2+^-sensor proteins, that significantly change their conformation as well as their solvation properties upon Ca^2+^ binding^19–21^, this aspect appears to be especially relevant. When a comparison of the hydrodynamic properties was made for a single Ca^2+^-sensor protein in the presence of several point mutations, a significant correlation between SPR, DLS, and SEC data was observed^21^, but comparisons cannot be easily extended to different proteins with different shapes and hydration shells due to different hydrophobic SAS. In conclusion, one should be extremely careful when using analytical SEC for assessing MW^SEC^ based on a standard of unrelated proteins and should always look for other independent estimates.

### Ca^2+^ and Mg^2+^ binding stabilize CIB2 tertiary structure

It was previously shown by NMR spectroscopy that saturation with either Mg^2+^ or Ca^2+^ makes CIB2 switch from a molten globule state to a structurally-folded state with very similar tertiary structure^7,8^. We performed limited proteolysis experiments to assess the sensitivity of CIB2 structural states to protease digestion. It is indeed known that binding of specific ligands may significantly affect the accessibility of proteins to proteases, thus providing useful information as to protein flexibility^22^.

**Figure 5:**
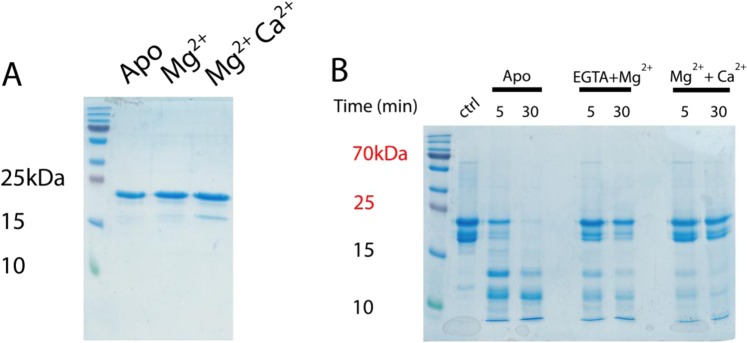
CIB2 limited proteolysis. (**A**) 10 µM CIB2 was incubated at 25 °C in the presence of 1 mM EDTA (Apo), 500 µM EGTA + 1 mM Mg^2+^ (Mg^2+^) and 1 mM Mg^2+^  + 2 mM Ca^2+^ for 10 minutes at 25 °C. Samples were boiled and loaded in a 18% SDS polyacrylamide gel. (**B**) Limited proteolysis was performed incubating trypsin with 25 µM CIB2 in the same concentrations of ions used for (**A**) (protein:enzyme ratio was 100:1). Samples were collected at different times, boiled and loaded on a 18% SDS-PAGE. Both gels run for 45–50 min, then they were Coomassie Blue-stained. The figure results from two separate gels, which have been reported in full length in Fig. S6.

Incubation of CIB2 with trypsin showed a clear time-dependent proteolytic pattern, which was found to depend on the cation-bound state (Fig. S2). We chose to further analyze the proteolytic pattern after 5 minutes and 30 minutes for apo-CIB2, as well as for the protein in the presence of 1 mM Mg^2+^ and after the further addition of 2 mM Ca^2+^ (Fig. 5).

Even without the addition of trypsin, CIB2 showed some propensity to degradation, as shown by the faint band appearing after 10 minutes at 25 °C under all tested conditions (Fig. 5A). The band was impossible to eliminate completely with the purification procedure and was observed previously before and after the cleavage of the His-tag^8^. MALDI experiments confirmed the presence of 16–17 kDa fragments (results not shown). The fact that the intensity of this band increased significantly in the time frame of the proteolysis experiments (Fig. 5B, control lane) supports its presumed nature.

Limited proteolysis clearly showed that after 30 min incubation with trypsin the native band of apo-CIB2 was completely lost, and several proteolytic fragments at lower molecular weight appeared. Interestingly, incubation with either Mg^2+^ or Ca^2+^ and Mg^2+^ permitted the detection of the native CIB2 band even after 30 min, although some smaller proteolytic fragments of comparable MW as to the ones for apo-CIB2 were clearly detected (Fig. 5B). Thus, Mg^2+^ and Ca^2+^ seem to exert a protective role in terms of accessibility to the protease, as a result of more rigid conformation, in line with previous observations^7,8^. It should be pointed, however, that some flexible regions remain even in the cation-complexed state, as clearly shown by the smaller MW bands.

In order to investigate the nature of the most flexible regions observed in cation-complexed CIB2, we performed 400 ns Molecular Dynamics (MD) simulations of Ca^2+^-CIB2 and computed the Root Mean-Squared Fluctuation index (RMSF) of Cα atoms. RMSF provides information as to the average displacement of selected atoms with respect to the initial position during the time course of the simulation, thus providing information on protein flexibility. The RMSF profile of Ca^2+^-saturated CIB2 (Fig. S3) is that of a somewhat flexible protein, as other WT Ca^2+^-sensors that underwent the same MD simulation procedure displayed significantly lower RMSF distributions^23–26^. The structural regions that showed higher flexibility are the pseudo EF1 motif (yellow segment, Figs 1 and S3), the loop linking EF3 and EF4 (orange and blue), the end of the exiting helix in EF4 and the C-terminus (blue and cyan). We speculate that these regions could be those that remain accessible to trypsin even when CIB2 is bound to Ca^2+^, due to their high intrinsic flexibility.

### Surface plasmon resonance points to monomeric CIB2 and highlights a low-affinity interaction with the membrane proximal segment of α7B integrin

SPR is a powerful and versatile technique to detect at real time biomolecular interactions, and it can be applied to study protein oligomerization, in particular dimerization, when care is taken to immobilize on the surface of a sensor chip low amounts of proteins under controlled conditions^27–29^. Two independent experiments were run following low levels of amine-coupling of CIB2 (130 RU and 226 RU, respectively) on the surface of a sensor chip. In no case was a signal detected when injecting uncoupled CIB2 in the 92 nM – 46 μM range in the presence of Ca^2+^ and Mg^2+^ (results not shown). Hence, SPR did not detect dimerization for CIB2 over a broad range of concentrations.

**Figure 6:**
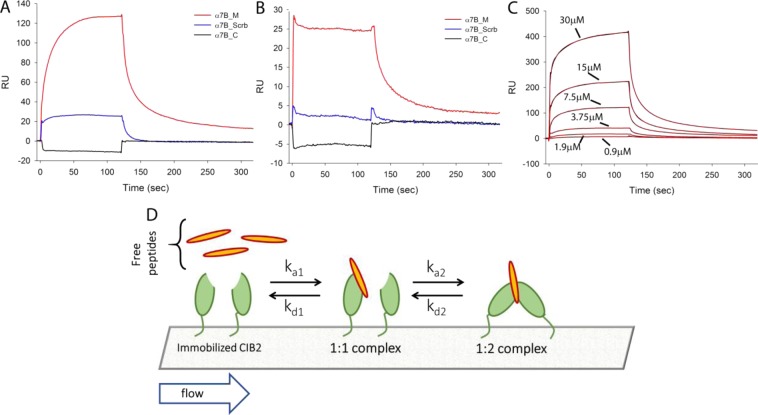
SPR measurements of CIB2-peptide interaction. (**A**) Either α7B_M, α7B_Scrb or α7B_C peptides (7.5 µM) were injected over His-CIB2 homogeneously immobilized by His-tag on the chip surface. (**B**) 30 µM of the same peptides were injected over untagged CIB2 previously immobilized on the surface of a COOH5 chip via amine coupling. (**C**) Increasing concentrations of α7B_M peptide were injected on His-CIB2 immobilized as in (**A**). Experimental data (black curves) were fitted to a bivalent analyte kinetic model (red curves). All the experiments were performed using 20 mM HEPES pH 7.5, 150 mM KCl, 1 mM DTT, 1 mM Mg^2+^, 2 mM Ca^2+^, 50 µM EDTA, 0.005% Tween as buffer. Two minutes injections were performed using a flow rate of 20 µL min^−1^, dissociations were followed for 200 seconds. (**D**) A possible scheme of CIB2-α7B_M interaction compatible with the kinetic model. The interaction of a CIB2 monomer immobilized on the chip (green) with a peptide (orange) can lead to the binding of another CIB2 molecule; all steps are fully reversible.

SPR was also used to study quantitatively the interaction of CIB2 with one of its established targets, namely the cytoplasmic domain of integrin α7B^3,7^. Three different peptides were used in the interaction experiments. A peptide corresponding to the membrane proximal segment of the cytoplasmic domain of α7B integrin (α7B_M), another peptide with the same amino acid content, but randomized sequence (α7B_Scrb) and a peptide covering the C-terminal region of the cytoplasmic domain of α7B integrin (α7B_C). Three different approaches were used. In a first approach, His-tagged CIB2 was initially coupled to the surface of a His-cap sensor chip (see Methods), thus ensuring homogeneous site-specific orientation, and then blocked via standard amine coupling. Near-UV CD spectroscopy (Fig. S4) confirmed that the His-tagged CIB2 had substantially the same tertiary structure of the untagged protein, and identically responded to Mg^2+^ and Ca^2+^. The minor differences in the spectra at the level of the phenylalanine band can be ascribed to the TEV-protease recognition site (ENLYFQ), uncleaved in the His-tagged protein. In a second approach, the α7B_M peptide was immobilized on the surface of a sensor chip by amine coupling at levels comparable to those for CIB2 in the former approach (2250 RU). In a third approach, we used the same low levels of CIB2 immobilization via amine coupling described in the experiments to probe CIB2 dimerization (100–200 RU) and flowed the three peptides. Examples of the obtained sensorgrams are reported in Fig. 6.

The second approach (direct peptide immobilization) led to very low signals and unreproducible results and was not pursued any further. Interestingly, both homogenous and site-specific coupling of CIB2 via its His- tag (approach 1) and heterogeneous coupling via non-specific amine coupling (approach 2) showed sensorgrams incompatible with a typical 1:1 Langmuir adsorption process. The two experiments showed clearly different association kinetics, and yet very similar dissociation kinetics incompatible with a monophasic exponential profile (Fig. 6A,B). In spite of such differences, which might reflect both the heterogeneity of the surface and the different chemistry of immobilization, interaction results were consistent with one another. Indeed, both experiments confirmed that α7B_M binds to CIB2 significantly stronger than the control peptide α7B_Scrb, which was shown to interact with low affinity also in MALDI experiments (Fig. 2B,C), where a band was detected in the same stoichiometric conditions, although with lower intensity. Moreover, the assays could not detect any interaction with the α7B_C peptide over the whole range of tested conditions. In this respect, our results are in line with those by Huang *et al*.^7^, who by fluorescence spectroscopy detected a much stronger interaction of CIB2 with the membrane-proximal region of the cytosolic tail of α7B integrin (corresponding to α7B_M), and a much weaker interaction with the C-terminal region, corresponding to the α7B_C peptide, at odds with Häger *et al*.^3^, who tested and detected interaction with the C-terminal region of α7B. It should be noted that both Huang *et al*.^7^ and Häger *et al*.^3^ used peptides from mouse integrin, while we used the corresponding human sequences, which in the case of α7B_C lacks the tryptophan residue present in the mouse sequence. It is not uncommon that fluorescence spectroscopy interaction studies based on the peptide intrinsic fluorescence (CIB2 has no tryptophan residue) reveal some non-specific binding, especially when a highly hydrophobic protein such as CIB2 is the ligand. Indeed, in our previous work we observed some non-specific signal when titrating CIB2 with α7B_Scrb, although fluorescence variation upon titration with α7B_M was much higher^8^. It cannot be excluded that a similar phenomenon occurred for murine α7B_C in the work by Häger *et al*.^3^. Our present results, based on accurate, label-free interaction studies clearly identify the region covered by α7B_M as the preferential binding site of CIB2 with α7B integrin.

SPR titrations of α7B_M over homogeneously coupled CIB2 (approach 1) gave very reproducible results and were performed both in the presence of 1 mM Mg^2+^ and in the co-presence of 1 mM Mg^2+^ and 2 mM Ca^2+^. Peptide concentration was varied in the 230 nM–30 μM range. An example of such titration is reported in Fig. 6C. As anticipated, sensorgrams could not fit to a 1:1 binding model, and the kinetic model that better fit the data was a bivalent analyte model (see Methods), in which one α7B_M peptide could bind to two CIB2 molecules. According to this scheme, binding of the peptide to the first CIB2 molecule would direct the binding to the second one, thus resulting in two sets of kinetic constants. Data fitting to this model was very good over the broad range of concentrations tested (Fig. 6C) and resulted in the kinetic parameters reported in Table 2. The same table also shows the results from steady state analysis, which allows an estimate of the total apparent affinity K_D_^appTOT^ accounting for avidity effects, as compared to that determined by the first binding event (one peptide to one CIB2 molecule, K_D_^app1^).

**Table 2:** Results from Surface Plasmon Resonance analysis of CIB2-α7B_M interaction.

	*k*_*a1*_ (M^−1^s^−1^)^a^	*k*_*a2*_(RU^−1^s^−1^)	*k*_*d1*_(s^−1^)	*k*_*d2*_(s^−1^)	K_D_^app1^ (µM)^b^	K_D_^appTOT^ (µM)^c^ (N = 28)
1 mM Mg^2+^ (N = 16)	(3.1 ± 0.1) × 10^4^	(1.9 ± 0.4) × 10^−3^	(3.6 ± 0.5) × 10^−1^	(2.0 ± 1.7) × 10^−2^	11.6 ± 1.8	210 ± 20
1 mM Mg^2+^ 2 mM Ca^2+^ (N = 15)	(1.6 ± 0.2) × 10^4^	(4.2 ± 0.5) × 10^−4^	(4.0 ± 0.3) × 10^−1^	(5.3 ± 0.5) × 10^−2^	25 ± 5	154 ± 8

^a^Kinetic constants were determined according to a bivalent analyte model as explained in Methods. N refers to the number of independently analyzed sensorgrams. Results refer to average ±  SEM.

^b^Apparent affinity for the first binding event, determined by the ratio *k*_*d1*_/*k*_*a1*._

^c^Apparent affinity constant accounting for avidity, determined by steady state analysis, measured by taking the maximum response RU^max^ for a series of 4 repetitions of 7 injections of α7B_M at increasing concentrations.

Our data suggest that CIB2 binds the α7B_M peptide with a relatively low affinity and that the formation of a protein-peptide complex may drive the binding of a second CIB2 molecule. Since CIB2 *per se* is monomeric, our data cannot exclude that binding of a target peptide induced dimerization of CIB2 on the chip, which would explain the high maximal response in terms of RU obtained in our binding experiments (Fig. 6C). Whether this process has a physiological meaning remains to be clarified. Interestingly, both the association process of α7B_M to the first CIB2 molecule, and then the association of the complex with a second CIB2 molecule occurred faster in the sole presence of Mg^2+^ compared to the co-presence of Ca^2+^ (2-fold for the first and 5-fold for the second binding event, respectively; Table 2). The dissociation process was not found to significantly depend on the cation conditions, the only significantly difference being the 2.5 times faster dissociation of the second CIB2 molecule in the co-presence of Mg^2+^ and Ca^2+^. In conclusion, the presence of Ca^2+^ seems to affect the recognition process of CIB2 and α7B in a complex way: it induces slower association and a slightly faster dissociation of the first CIB2-peptide complex, thus resulting in roughly half affinity compared to Mg^2+^ alone (K_D_^app1^ = 25 μM vs 11.6 μM), however when the whole binding process is considered avidity comes into play and the apparent affinity in the presence of both Mg^2+^ and Ca^2+^ (K_D_^appTOT^ = 154 μM) seems to be slightly higher compared to that measured in the sole presence of Mg^2+^ (K_D_^appTOT^ = 210 μM).

To our knowledge, no other kinetic study was performed so far for CIB2-target recognition, the only possible comparison is thus with previous SPR experiments performed with the homolog CIB1 protein interacting with a peptide from αIIb integrin^30^, which according to a 1:1 binding model revealed a very similar apparent affinity (K_D_^app^ = 12 μM) without significant dependency on Ca^2+^. It should be noted, however, that sequence and presumably structural/functional features of CIB2 could significantly differ from those of CIB1^8^ and that even for the same system different studies reported quite different affinities, probably due to the heterogeneous set of experimental techniques used (see ref.^31^ for a review).

### Interaction between CIB2 and α7B_M and structural rearrangement

SPR data suggest a relatively low affinity interaction of CIB2 with the α7B_M peptides, and results from MALDI experiments seem to support this finding (Fig. 2B).

Near-UV CD spectroscopy was used to study the structural effects of the binding of α7B_M peptide to CIB2 in the presence and in the absence of cations (Fig. S5). The isolated peptide was unfolded in all the tested cases, but interestingly, when incubated with CIB2 some structural effects could be observed both for 1:1 and 2:1 peptide:protein conditions. For apo-CIB2, although the spectrum was still compatible with a molten globule state (Fig. S5A), peptide induced a finer spectrum in the phenylalanine and tyrosine bands, thus suggesting interaction between CIB2 and α7B_M even in the absence of cations. When the incubation was done in the presence of Mg^2+^ as well as in the co-presence of Ca^2+^, the intensity of the resulting spectrum was lower compared to that of CIB2 without the peptide (Fig. S5A,B) and the decrease was proportional to the stoichiometric peptide:protein ratio, being especially apparent for the 2:1 ratio. A closer inspection showed mild alterations of the spectrum especially of the level of the phenylalanine band, with some smoothening of the tyrosine region in the case of incubation with only Mg^2+^, however no clear signal was visible in the typical 285–305 nm tryptophan region. Since α7B has one tryptophan and CIB2 has none, the lack of signal following incubation suggests that the peptide did not acquire a defined structure upon interaction, at least not completely. If this was the case, the effect would have been likely visible, such as in the case of calmodulin-target interaction^32,33^. The decrease in the intensity of near-UV CD spectrum, more prominent for the case of Mg^2+^ alone, could then be attributed to some global effect such as increased flexibility and/or solvent accessibility of CIB2 upon interaction with the peptide, or even a dimerization of CIB2 induced by the bound peptide, which would lead to enhanced exposure of aromatic residues to the solvent.

## Conclusions

Ca^2+^-binding proteins belonging to the EF-hand superfamily may exist in a variety of conformational states^34^ and many were found to form dimers depending on their cation-loading state and concentration^35^. CIB2 has been found to form dimers in previous studies, where it was fused with GFP^11,12^ and tdTomato fluorescent protein^11^ and studied in FRET and co-immuno precipitation experiments. Our previous work with untagged purified CIB2 also concluded that CIB2 is dimeric^8^. We now provide new lines of evidence that allow us to reconsider some of the previous findings in the light of MS and SPR data. Based on our extensive analysis performed in a variety of conditions and over a broad range of concentrations we can conclude that CIB2 is *per se* monomeric, but presents uncommon hydrodynamic properties likely due to the high content of hydrophobic solvent accessible surface, which may lead to the erroneous appearance of a dimer. This feature, together with the possible interaction between fusion-proteins or tags and the highly hydrophobic surface of CIB2 calls for special care when assessing its oligomeric state.

Interaction with α7B occurs in the region proximal to the membrane and not close to the C-terminus of the cytosolic domain of the integrin. The recognition between CIB2 and the α7B target is kinetically favored in the presence of physiological Mg^2+^ and in the absence of Ca^2+^ and it might induce binding of another CIB2 molecule. This hypothesis seems to be in line with our previous fluorescence data where CIB2 was shown to interact with the target peptide with a 2:1 stoichiometry^8^. However, CIB2 *per se* binds a single peptide as a monomer, and the complex could then induce CIB2 dimerization, where the dimer is bridged by the target. Considering the largely unknown biological function of CIB2 and that the homolog protein CIB1 allows a high degree of promiscuity in target recognition^31^, this putative mechanism of target-induced dimerization might play a role in integrin signaling. Whether this intriguing scenario may have any physiological consequence needs to be further investigated.

## Methods

### Protein and peptide preparation and purification

Recombinant human CIB2 was obtained exactly as explained in our previous work^8^. Untagged protein was used for all experiments except for one case of SPR interaction where the TEV protease recognition site and the Histag were kept. Human CaM and bovine Rec were expressed and purified as described in previous work^36,37^.

Peptides were purchased from Genscript and had the following sequence: α7B_M LLLWKMGFFKRAKHPE (HPLC purity = 96.7%), α7B_Scrb KEFWGLHAKPRLKLMF (HPLC = 96.3%), α7B_C LAADGHPELGPDGHPGPGTA (HPLC = 98.8%). Both α7B_M and α7B_Scrb were C-terminal amidated and N-terminal acetylated, while α7B_C was N-terminal acetylated. All peptides were initially resuspended in pure water and then diluted at final concentration in the working buffer.

### Cross-linking reactions

CIB2 samples were cross-linked with DSBU in the presence of peptide α7B_M or α7B_Scrb as described in ref.^14^ Briefly, 10 µM CIB2 was diluted in 20 mM HEPES, pH 7.5, 1 mM DTT, 1 mM Mg^2+^ and 2 mM Ca^2+^ and incubated with increasing peptide concentrations (5 µM, 10 µM, and 20 µM) for 10 min at room temperature. Then, 50-fold molar excess of DSBU was added and the reactions were conducted for 20 min at room temperature. As control, CIB2 was treated with DSBU without peptide. Reactions were blocked by adding ammonium bicarbonate to a final concentration of 20 mM.

### Mass spectrometry

Cross-linked CIB2 samples were desalted via ZipTip C4 tips (Millipore) before MALDI-TOF-MS analysis (Ultraflex III MALDI-TOF/TOF mass spectrometer, Bruker Daltonik, Bremen) was conducted using sinapinic acid as matrix. For further details see Supplementary Methods. Baseline subtraction and spectra smoothing were performed.

For native ESI-MS analyses (High-Mass Q-TOF II mass spectrometer, Waters/MSVision), CIB2 was prepared in 200 mM ammonium acetate, pH 6.8.

### Analytical size exclusion chromatography

The molecular weight (MW) of the proteins was determined before and after DSBU-modification as previously described^7^ in the same buffer (20 mM HEPES, pH 7.5, 1 mM DTT, 1 mM Mg^2+^ and 2 mM Ca^2+^). Protein concentration was determined by Bradford Assay and was found to be 113 µM for CaM and between 70–80 µM for CIB2 and Rec. Standard calibration curve for MW estimation was the same used in our previous study^8^.

### DLS experiments

DLS measurements were performed using Zetasizer Nano-S (Malvern Instruments) at 37 °C by setting the same parameters for hydrodynamic diameter estimation as in ref.^26^. The estimation was based on 15–30 measurements, each consisting of at least 13 repetitions; data in Table 1 are reported as mean ± standard error of mean (SEM). DLS samples for each protein are the same collected in the Analytical Size Exclusion Chromatography. Protein concentration was measured by Bradford Assay for Rec, CaM and CIB2 and was found to be 11 μM, 7.4 μM and 1.8 μM respectively.

### Circular dichroism spectroscopy

CIB2 tertiary structure was investigated by using a Jasco J-710 spectropolarimeter equipped with a Peltier type cell holder as previously described^37^. Briefly: near-UV (250–320 nm) CD spectra of 20 μM of sole CIB2 or CIB2/α7B_M complexes (1:1 and 1:2 ratios) were collected in the presence of 500 μM EGTA and after sequential additions of 1 mM Mg^2+^ and 2 mM free Ca^2+^. A 1 cm quartz cuvette was used, temperature was set at 37 °C, integration time and data pitch were set to 4 s and 1 nm, respectively. Each spectrum represents the average of 5 accumulations.

### Limited proteolysis experiments

Limited proteolysis experiments were performed incubating 25 µM CIB2 in the presence of Trypsin (Sigma) in a ratio 1:100 enzyme:CIB2 at 25 °C. Three conditions were tested: 1 mM EDTA, 500 µM EGTA + 1 mM Mg^2+^ and 1 mM Mg^2+^  + 2 mM Ca^2+^. Time-resolved proteolysis patterns are reported in Supplementary Materials (Fig. S2), results shown in Fig. 5 refer to reactions stopped after 5 min and 30 min. Proteolysis products were loaded on a 15% or 18% SDS gel, as indicated.

### Molecular dynamics simulations

The homology model of Ca^2+^-bound human CIB2 was built using the X-ray structure (resolution: 1.99 Å) of human CIB1 as a template (PDB entry: 1XO5)^2^, which shares 39% sequence identity with CIB2. Modeling details are provided in our previous work^8^. The PDB file of the model is available upon request for research purposes. MD simulations of Ca^2+^-bound CIB2 were performed using GROMACS 2016.1 simulation package^38^ and CHARMM36m^39^ all-atom force field (system size 47780 atoms). All simulation details were the same as in previous work^25,40^. Root-Mean Square Fluctuation of Cα along the 400-ns trajectory was calculated with respect to the equilibrated structure using “gmx rmsf” function included in GROMACS 2016.1 as previously explained^23^.

### Hydrodynamic diameter estimation

Estimation of the hydrodynamic diameter was performed using Hydropro^16^ on the structure of bovine Rec after removal of the GRK1 peptide^41^, and human CIB2^8^. Details on the preparation of human CaM structure as well as on the determination of the hydrodynamic diameter are given in the Supplementary Information.

### Surface plasmon resonance

Details of the protein and peptide immobilization procedures and experimental settings are elucidated in the Supplementary Information.

Kinetic data were fitted according to a bivalent analyte model, were a CIB2 monomer (L) can bind an α7B_M peptide (A), and the resulting complex (AL) can bind to a second CIB2 molecule (AL_2_), with all processes being possibly reversible, as follows:

$$\begin{array}{lll}A+L & \mathop{\to }\limits^{{k}_{a1}} & AL\\ AL & \mathop{\to }\limits^{{k}_{d1}} & A+L\\ AL+L & \mathop{\to }\limits^{{k}_{a2}} & A{L}_{2}\\ A{L}_{2} & \mathop{\to }\limits^{{k}_{d2}} & AL+\,L\end{array}$$

The following set of ordinary differential equations can be numerically solved to determine the kinetic parameters:

$$\begin{array}{rcl}\frac{d[L]}{dt} & = & -(k{a}_{1}\cdot [A]\cdot [L]-k{d}_{1}\cdot [AL])-(k{a}_{2}\cdot [AL]\cdot [L]-k{d}_{2}\cdot [A{L}_{2}])\\ \frac{d[AL]}{dt} & = & (k{a}_{1}\cdot [A]\cdot [L]-k{d}_{1}\cdot [AL])-(k{a}_{2}\cdot [AL]\cdot [L]-k{d}_{2}\cdot [A{L}_{2}])\\ \frac{d[A{L}_{2}]}{dt} & = & k{a}_{2}\cdot [AL]\cdot [L]-k{d}_{2}\cdot [A{L}_{2}]\\ L(0) & = & {R}_{max};AL(0)=A{L}_{2}(0)=0\end{array}$$

where [A](t) is the molar concentration of α7B_M peptide at time t; L is the available CIB2 immobilized; [AL](t) is the concentration of the CIB2-α7B_M complex at time t; [AL_2_](t) is the concentration of the CIB2-α7B_M-CIB2 complex at time t; *k*_*a1*_ is the association rate constant for the CIB2-α7B_M complex (M^−1^s^−1^); *k*_*d1*_ is the dissociation rate constant for the CIB2-α7B_M complex (s^−1^); *k*_*a2*_ is the association rate constant for the CIB2-α7B_M-CIB2 complex (RU^−1^s^−1^), driven by the first association; *k*_*d2*_ is the dissociation rate constant for the CIB2-α7B_M-CIB2 complex (s^−1^).

Data fitting was performed using the software QDAT (Pall FortéBio, LCC 2016).

## Supplementary information

Supplementary information

## References

[1] Seki N (1999). Structure, expression profile and chromosomal location of an isolog of DNA-PKcs interacting protein (KIP) gene. Biochim Biophys Acta.

[2] Gentry HR (2005). Structural and biochemical characterization of CIB1 delineates a new family of EF-hand-containing proteins. J Biol Chem.

[3] Hager M (2008). Cib2 binds integrin alpha7Bbeta1D and is reduced in laminin alpha2 chain-deficient muscular dystrophy. J Biol Chem.

[4] Godinho-Santos A, Hance AJ, Goncalves J, Mammano F (2016). CIB1 and CIB2 are HIV-1 helper factors involved in viral entry. Sci Rep.

[5] Blazejczyk M (2009). Biochemical characterization and expression analysis of a novel EF-hand Ca^2+^ binding protein calmyrin2 (Cib2) in brain indicates its function in NMDA receptor mediated Ca^2+^ signaling. Arch Biochem Biophys.

[6] Zhu W (2017). CIB2 Negatively Regulates Oncogenic Signaling in Ovarian Cancer via Sphingosine Kinase 1. Cancer Res.

[7] Huang H, Bogstie JN, Vogel HJ (2012). Biophysical and structural studies of the human calcium- and integrin-binding protein family: understanding their functional similarities and differences. Biochem Cell Biol.

[8] Vallone R, Dal Cortivo G, D'Onofrio M, Dell'Orco D (2018). Preferential Binding of Mg(2+) Over Ca(2+) to CIB2 Triggers an Allosteric Switch Impaired in Usher Syndrome Type 1J. Front Mol Neurosci.

[9] Wang Y (2017). Loss of CIB2 Causes Profound Hearing Loss and Abolishes Mechanoelectrical Transduction in Mice. Front Mol Neurosci.

[10] Patel K (2015). A Novel C-Terminal CIB2 (Calcium and Integrin Binding Protein 2) Mutation Associated with Non-Syndromic Hearing Loss in a Hispanic Family. PLoS One.

[11] Riazuddin S (2012). Alterations of the CIB2 calcium- and integrin-binding protein cause Usher syndrome type 1J and nonsyndromic deafness DFNB48. Nat Genet.

[12] Giese APJ (2017). CIB2 interacts with TMC1 and TMC2 and is essential for mechanotransduction in auditory hair cells. Nat Commun.

[13] Sinz A (2018). Cross-Linking/Mass Spectrometry for Studying Protein Structures and Protein-Protein Interactions: Where Are We Now and Where Should We Go from Here?. Angew Chem Int Ed Engl.

[14] Iacobucci C (2018). A cross-linking/mass spectrometry workflow based on MS-cleavable cross-linkers and the MeroX software for studying protein structures and protein-protein interactions. Nat Protoc.

[15] Piotrowski C, Sinz A (2018). Structural Investigation of Proteins and Protein Complexes by Chemical Cross-Linking/Mass Spectrometry. Adv Exp Med Biol.

[16] Ortega A, Amoros D, Garcia de la Torre J (2011). Prediction of hydrodynamic and other solution properties of rigid proteins from atomic- and residue-level models. Biophys J.

[17] Myers WK (2013). Double electron-electron resonance probes Ca(2)(+)-induced conformational changes and dimerization of recoverin. Biochemistry.

[18] La Verde V, Dominici P, Astegno A (2017). Determination of Hydrodynamic Radius of Proteins by Size Exclusion Chromatography. Bio-Protocol.

[19] Dell'Orco D, Muller M, Koch KW (2010). Quantitative detection of conformational transitions in a calcium sensor protein by surface plasmon resonance. Chem Commun (Camb).

[20] Dell'Orco D, Sulmann S, Linse S, Koch KW (2012). Dynamics of conformational Ca^2+^ -switches in signaling networks detected by a planar plasmonic device. Anal Chem.

[21] Sulmann S, Dell'Orco D, Marino V, Behnen P, Koch KW (2014). Conformational changes in calcium-sensor proteins under molecular crowding conditions. Chemistry.

[22] Fontana, A. *et al*. Probing protein structure by limited proteolysis. *Acta Biochim Pol* **51**, 299-321, 035001299 (2004). 15218531

[23] Marino V (2018). A novel p.(Glu111Val) missense mutation in GUCA1A associated with cone-rod dystrophy leads to impaired calcium sensing and perturbed second messenger homeostasis in photoreceptors. Hum Mol Genet.

[24] Marino V, Scholten A, Koch KW, Dell'Orco D (2015). Two retinal dystrophy-associated missense mutations in GUCA1A with distinct molecular properties result in a similar aberrant regulation of the retinal guanylate cyclase. Hum Mol Genet.

[25] Marino V, Sulmann S, Koch KW, Dell'Orco D (2015). Structural effects of Mg(2)(+) on the regulatory states of three neuronal calcium sensors operating in vertebrate phototransduction. Biochim Biophys Acta.

[26] Vocke F (2017). Dysfunction of cGMP signalling in photoreceptors by a macular dystrophy-related mutation in the calcium sensor GCAP1. Hum Mol Genet.

[27] Anggayasti WL, Mancera RL, Bottomley S, Helmerhorst E (2016). The effect of physicochemical factors on the self-association of HMGB1: A surface plasmon resonance study. Biochim Biophys Acta.

[28] Anggayasti WL, Mancera RL, Bottomley S, Helmerhorst E (2016). Optimization of surface plasmon resonance experiments: Case of high mobility group box 1 (HMGB1) interactions. Anal Biochem.

[29] Singh A (2015). Analysis of AKAP7gamma Dimerization. J Signal Transduct.

[30] Vallar L (1999). Divalent cations differentially regulate integrin alphaIIb cytoplasmic tail binding to beta3 and to calcium- and integrin-binding protein. J Biol Chem.

[31] Leisner TM, Freeman TC, Black JL, Parise LV (2016). CIB1: a small protein with big ambitions. FASEB J.

[32] Astegno A, La Verde V, Marino V, Dell'Orco D, Dominici P (2016). Biochemical and biophysical characterization of a plant calmodulin: Role of the N- and C-lobes in calcium binding, conformational change, and target interaction. Biochim Biophys Acta.

[33] Astegno A (2014). Structural plasticity of calmodulin on the surface of CaF2 nanoparticles preserves its biological function. Nanoscale.

[34] Yap KL, Ames JB, Swindells MB, Ikura M (1999). Diversity of conformational states and changes within the EF-hand protein superfamily. Proteins.

[35] Ames JB (2018). Dimerization of Neuronal Calcium Sensor Proteins. Front Mol Neurosci.

[36] Dal Cortivo G (2018). Luminescent and paramagnetic properties of nanoparticles shed light on their interactions with proteins. Sci Rep.

[37] Marino V, Astegno A, Pedroni M, Piccinelli F, Dell'Orco D (2014). Nanodevice-induced conformational and functional changes in a prototypical calcium sensor protein. Nanoscale.

[38] Abraham MJ (2015). GROMACS: High performance molecular simulations through multi-level parallelism from laptops to supercomputers. SoftwareX.

[39] Huang J (2017). CHARMM36m: an improved force field for folded and intrinsically disordered proteins. Nat Methods.

[40] Marino V, Dell'Orco D (2016). Allosteric communication pathways routed by Ca(2+)/Mg(2+) exchange in GCAP1 selectively switch target regulation modes. Sci Rep.

[41] Marino V, Dell'Orco D (2019). Evolutionary-Conserved Allosteric Properties of Three Neuronal Calcium Sensor Proteins. Front Mol Neurosci.

