# Supplementary material for: Oligomeric state, hydrodynamic properties and target recognition of human Calcium and Integrin Binding protein 2 (CIB2)
Source: Sci Rep. 2019 Oct 21;9:15058. doi: 10.1038/s41598-019-51573-3 (PMC6803640; doi:10.1038/s41598-019-51573-3)
Supplement: Supplementary file 1 — Supplementary information [file 41598_2019_51573_MOESM1_ESM.pdf]

Supporting Information

Oligomeric state, hydrodynamic properties and target recognition of human Calcium and Integrin Binding protein 2 (CIB2)

by

Giuditta Dal Cortivo, Valerio Marino, Claudio Iacobucci, Rosario Vallone, Christian Arlt, Anne Rehkamp, Andrea Sinz and Daniele Dell'Orco

Supplementary Methods

Cross-linking reactions and Mass Spectrometry

Cross-linking reactions were performed with 10 µM CIB2 (in 20 mM HEPES pH 7.5, 150 mM KCl, 1 mM DTT, 1 mM Mg^{2+}, 2 mM Ca^{2+}) and increasing concentrations of peptides α7B_M and α7B_Scrb, ranging from 0.5:1 to 2:1 peptide:protein ratios. After incubating the samples for 10 minutes at room temperature, DSBU (freshly prepared solution, dissolved in neat DMSO) was added to the mixture at 50-fold molar excess compared to CIB2 concentration. As the final volume of the reaction mixture was 50 µL, 1 µL of 25 mM DSBU was added to each sample. After 20 minutes, the cross-linking reactions were quenched by adding ammonium bicarbonate to a final concentration of 20 mM. Samples were incubated for 10 minutes before SDS-PAGE analysis was performed (data not shown). Samples for MALDI-TOF-MS measurements were desalted by ZipTip C4 (Millipore) and desalted protein was eluted with 70% (v/v) acetonitrile / 1% (v/v) formic acid. Each sample was mixed with a saturated solution of sinapinic acid (in 50% (v/v) methanol / 0.1% (v/v) TFA) at 1:1 ratio and spotted onto the MALDI target.

Hydrodynamic diameter estimation

Since human CaM (pdb 1CLL)[1] was lacking the first 4 residues at the N-term, they were modeled using pdb structure 4e53 as template.[2] After superimposition of the Cα of residues 4-8, residues 1-7 of 4e53 were fused with residues 8-148 from 1CLL. Analogously, the missing Lys 149 at the C-term was modeled using the pdb structure 4djc[3] as a template by fusion of residues 146-149 of 4djc with residues 1-145 of 1CLL after superimposition of residues 146-148 Cα.

Hydrodynamic diameter was estimated using default values for radius of atomic elements (2.84 Å), solvent density (1 g/ml) and solvent viscosity (0.01 poise), temperature was set to 25 °C and the mode was set to shell-model from atomic level. Specific volume for each protein structure was calculated

by summing the volume of each residue and correcting the final volume for electrostriction as explained in Ref.[4], therefore the specific volume for Rec, CaM and CIB2 were 0.712, 0.694 and 0.715 cm^3/g respectively. The molecular weight used for the estimation of hydrodynamic diameter was calculated based on the effective protein sequence of each pdb file using Protein Calculator 3.4 (http://protcalc.sourceforge.net/).

Surface plasmon resonance analysis

A SensiQ Pioneer surface plasmon resonance instrument (ForteBio Pall) was employed for the investigation of the CIB2 dimerization and for the real-time monitoring of its interaction with selected peptides. For studies of on-chip CIB2 dimerization, HBS buffer (20 mM HEPES, pH 7.5, 150 mM KCl, 1 mM $MgCl_2$, 50 µM EDTA, 0.005% Tween 20), was used both as immobilization and running buffer, with the addition of fresh 1 mM DTT just before each experiment.

CIB2 was immobilized on the surface of a COOH5 sensor chip (FortéBio Pall) coated with a carboxylated polysaccharide hydrogel spacer. Carboxyl groups were mobilized with sequential injections (5 µl min^{-1} flow rate) of 60 µl of 10 mM H_3PO_4, 60 µl HBS, 60 µl 10 mM NaOH and 2 × 60 µl HBS. The sensor chip surface was then activated with a 7 min injection of a mixture of 10 mM *N*-hydroxysuccinimide (NHS) and N-ethyl-N′-(dimethylaminopropyl)carbodiimide (EDC) at a flow rate of 5 µl min^{-1}. Previously frozen samples of untagged CIB2 in HBS were thawed and diluted to a final concentration of 50 nM in Na-acetate buffer (pH 3.1), 1.52 pKa units below its isoelectric point. Amine coupling of CIB2 was achieved with a slow and prolonged sequential injection of protein at flow rate of 10 µl min^{-1} leading to a final amount of 130 RU in one case and 226 RU in another experiment (1 RU = 1 pg mm^{-2}). The surface was blocked via injection of 70 µl 1M ethanolamine hydrochloride-NaOH, pH 8.5. In all SPR experiments, one of the three flow cells was activated and then blocked without protein immobilization, and was used as a reference. CIB2 was diluted to final concentrations of 92 nM – 46 µM and injected at a flow rate of 20 µl min^{-1} (2 min injection) resulting in all cases in flat sensorgrams, except for some non-specific bulk effect.

In the experiments concerning the interaction with peptides, the same chips with amine-coupled CIB2 were used and the three peptides α7B_M, α7B_Scrb and α7B_C were diluted to a final concentration of 7.5 – 30 µM in HBS and injected in the same conditions (20 µl min^{-1}, 2 min injection); experiments were repeated 4 times.

CIB2 was also homogeneously immobilized at higher levels using the capture-coupling technique, where His-tagged CIB2 is first site-specifically driven on the surface of a His-Cap sensor chip (FortéBio Pall), based on a three-dimensional non-dextran hydrogel polymer modified with Nitrilotriacetic acid (NTA) functional groups that chelate Nickel ions for the selective capture of 6X

His-tagged proteins, and then blocked via amine coupling as explained above. Preparation of the sensor chip and immobilization followed the instructions from the manufacturer and resulted in approximately 2250 RU of CIB2 immobilized. In binding experiments, peptide concentration was varied in the 230 nM- 30 µM range for both α7B_M, and α7B_C, and experiments were repeated 4 times, both with the HBS buffer described above (containing 1 mM $MgCl_2$) and with further addition of 2 mM $CaCl_2$. Flow rate was set to 20 µl min^{-1} in all binding experiments, and the dissociation process was followed for 240 s, resulting in complete protein-peptide dissociation.

Supplementary figures

Figure S1

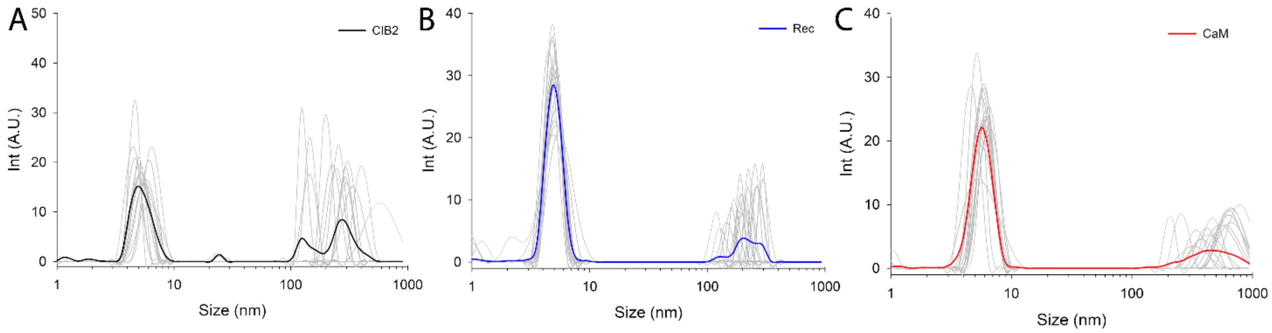

Figure S1: DLS measurements of the SEC collected peaks. CIB2 band A_2 (A), Rec (B) and CaM (C). Each data set consisted of 15-30 measurements (grey lines), with 13-15 repetitions each. Colored lines are referred to the average distribution. Temperature was set to 37°C and samples were equilibrated for 2 min before measurements.

Figure S2

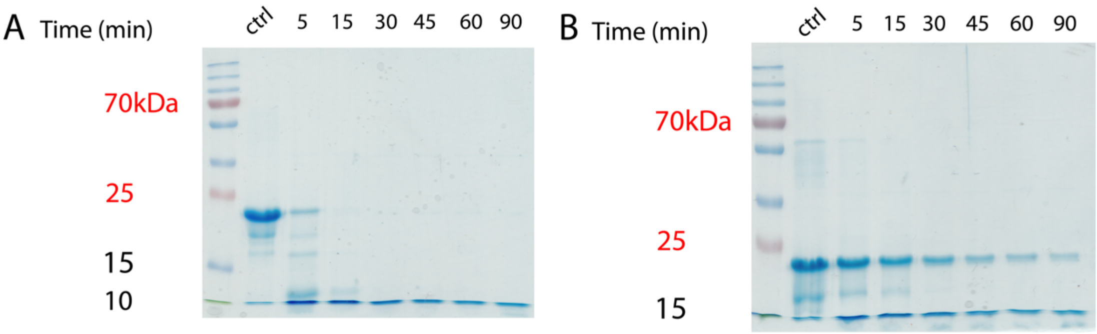

Figure S2: Time-resolved proteolysis experiments. 25 µM CIB2 was incubated with Trypsin (1:100 enzyme:CIB2 ratio) in the presence of 1 mM EDTA (A) or 500 µM EGTA + 1 mM Mg^{2+} (B) for 90 minutes, samples were collected at different times. Temperature was set to 25°C and CIB2 without enzyme was used as control. Reaction products were loaded on a 15% SDS-PAGE.

Figure S3

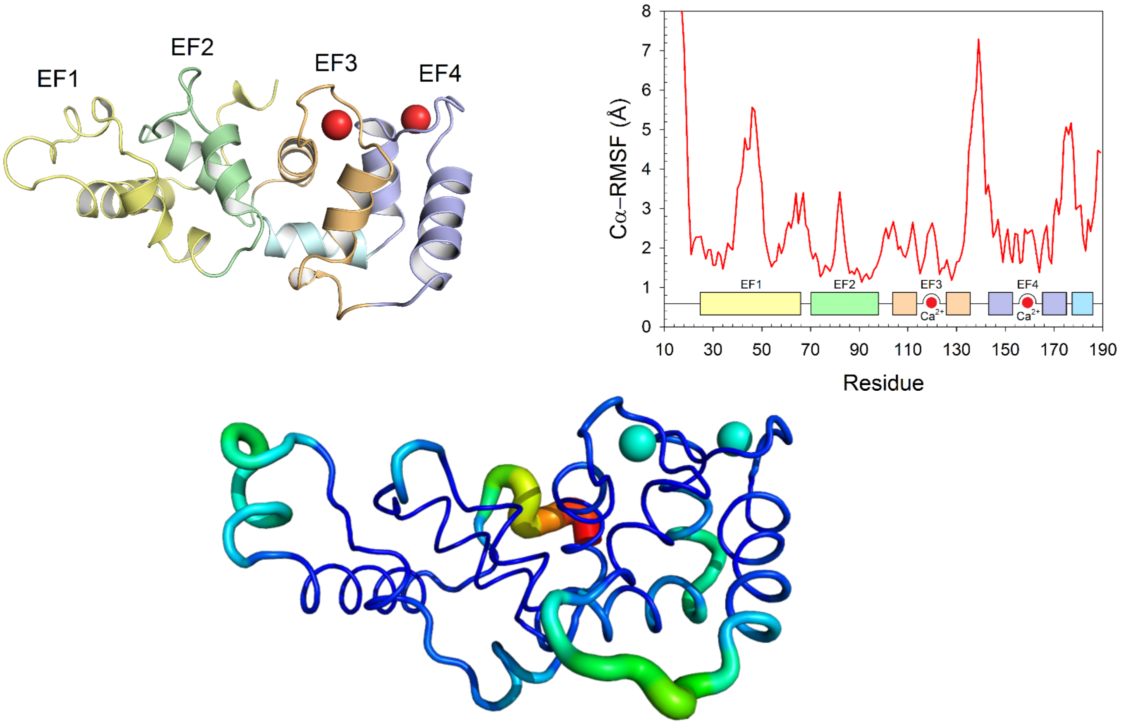

Figure S3: Analysis of MD simulations. Three-dimensional structure of the homology model of Ca^{2+}-bound human CIB2 (top left), protein secondary structure is shown as cartoons, EF1 is colored in yellow, EF2 is colored in green, EF3 is colored in orange, EF4 is colored in blue, C-term helix is colored in cyan and Ca^{2+} ions are shown as red spheres. Root-Mean Square Fluctuation of Cα (top right) calculated over 400 ns MD simulations, inset shows protein secondary structure where EF hands and Ca^{2+} ions are colored according to the left panel. Projection of the Cα-RMSF (bottom) on the three-dimensional structure of CIB2, Ca^{2+} ions are shown as spheres, protein is shown as cartoons, both are colored in a blue-to-red scale according to their Cα-RMSF, ranging from 1.1 to 13.9 Å. Thickness of the cartoon is also proportional to the Cα-RMSF.

Figure S4

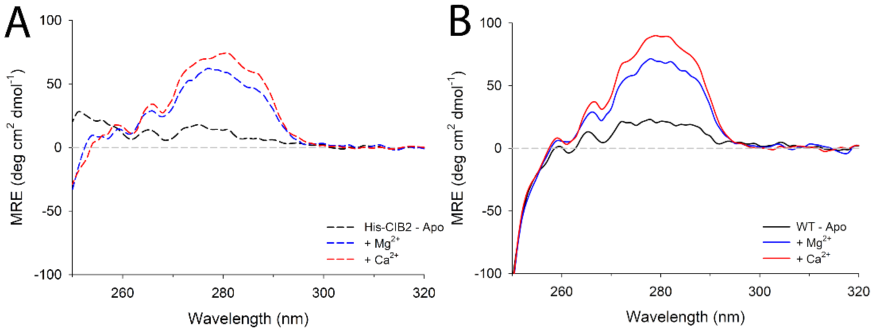

Figure S4: Structural comparison of His-CIB2 (A) and CIB2 (B). Near-UV CD spectra (250-320 nm) of 20-30 μM CIB2 were collected at 37°C in a quartz cuvette with 1 cm length path. Sequential additions of 500 μM EGTA (black), 1 mM Mg^{2+} (blue) and 2 mM Ca^{2+} (red) were performed. Near-UV CD spectra of the buffer (20 mM HEPES pH 7.5, 150 mM KCl, 1 mM DTT) was considered as blank and subtracted. Each spectrum represents the mean of 5 accumulations. Data were normalized according to the protein concentration and the number of residues (Molar Residue Ellipticity, MRE).

Figure S5

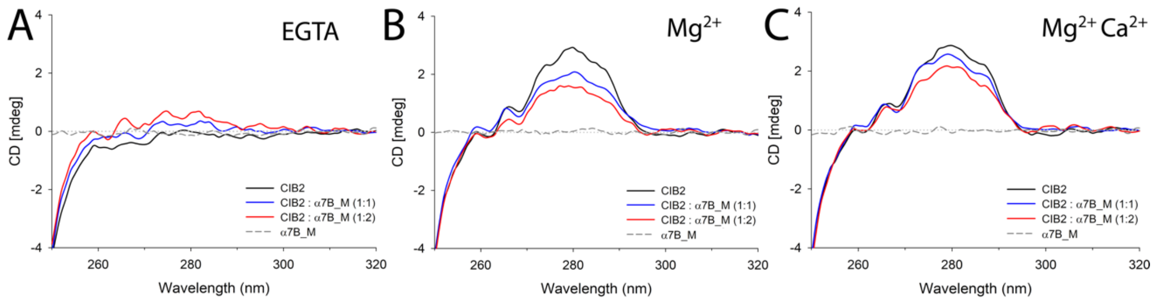

Figure S5: Near-UV CD spectra of CIB2 in the presence of different stoichiometric ratios of alpha7B_M peptide. Near-UV CD spectra (250-320nm) of 20 µM CIB2 in the absence of peptide (black lines) and in the presence of two different protein/peptide ratios (1:1 blue lines; 1:2 red lines) collected at 37°C in the presence of 500 µM EGTA (A) and after sequential additions of 1 mM Mg^{2+} (B) and 2 mM free Ca^{2+} (C). The spectrum of alpha7B_M peptide in the absence of CIB2 is shown in dashed grey lines.

Figure S6

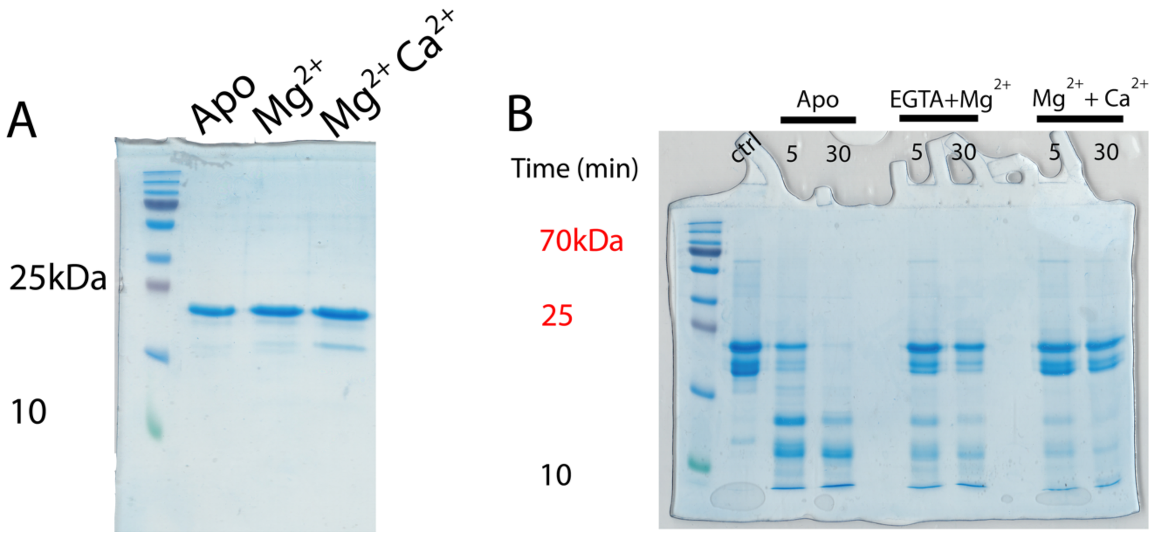

Figure S6. CIB2 limited proteolysis. A) 10 µM CIB2 was incubated at 25°C in the presence of 1 mM EDTA (Apo), 500 µM EGTA + 1 mM Mg^{2+} (Mg^{2+}) and 1 mM Mg^{2+} + 2 mM Ca^{2+} for 10 minutes at 25°C. Samples were boiled and loaded in a 18% SDS polyacrylamide gel. B) Limited proteolysis was performed incubating trypsin with 25 µM CIB2 in the same concentrations of ions used for (A) (protein:enzyme ratio was 100:1). Samples were collected at different times, boiled and loaded on a 18% SDS-PAGE. Both gels run for 45-50 min, then they were Coomassie Blue-stained. Uncropped

Supplementary References

1. Chattopadhyaya, R.; Meador, W. E.; Means, A. R.; Quiocho, F. A., Calmodulin structure refined at 1.7 A resolution. *J Mol Biol* **1992,** *228* (4), 1177-92.
2. Kumar, V.; Chichili, V. P.; Zhong, L.; Tang, X.; Velazquez-Campoy, A.; Sheu, F. S.; Seetharaman, J.; Gerges, N. Z.; Sivaraman, J., Structural basis for the interaction of unstructured neuron specific substrates neuromodulin and neurogranin with Calmodulin. *Sci Rep* **2013,** *3*, 1392.
3. Sarhan, M. F.; Tung, C. C.; Van Petegem, F.; Ahern, C. A., Crystallographic basis for calcium regulation of sodium channels. *Proc Natl Acad Sci U S A* **2012,** *109* (9), 3558-63.
4. Harpaz, Y.; Gerstein, M.; Chothia, C., Volume changes on protein folding. *Structure* **1994,** *2* (7), 641-9.
